# National practice patterns and direct medical costs for prostate cancer in Korea across a 10 year period: a nationwide population-based study using a national health insurance database

**DOI:** 10.1186/s12913-019-4218-7

**Published:** 2019-06-24

**Authors:** Ho Won Kang, Seok-Joong Yun, Jae Il Chung, Hoon Choi, Jae Heon Kim, Ho Song Yu, Yun-Sok Ha, In-Chang Cho, Hyung Joon Kim, Hyun Chul Chung, Jun Sung Koh, Wun-Jae Kim, Jong-Hyock Park, Ji Youl Lee, So-Young Kim

**Affiliations:** 1 Department of Urology, Chungbuk National University Hospital, College of Medicine, Chungbuk National University, Cheongju, Korea; 2 Department of Urology, Inje University Busan Paik Hospital, Busan, Korea; 3 Department of Urology, Korea University Ansan Hospital, Korea University College of Medicine, Ansan, Korea; 4 Department of Urology, Soonchunhyang University Hospital, Seoul, Korea; 5 Department of Urology, Chonnam National University Medical School, Gwangju, Korea; 6 Department of Urology, School of Medicine, Kyungpook National University, Daegu, Korea; 7 Department of Urology, National Police Hospital, Seoul, Korea; 8 Department of Urology, Konyang University College of Medicine, Daejeon, Korea; 9 Department of Urology, Wonju College of Medicine, Yonsei University, Wonju, Korea; 10 Department of Urology, Seoul St. Mary’s Hospital, The Catholic University of Korea School of Medicine, 222 Banpo-daero, Seocho-gu, Seoul, 06591 Korea; 11 Department of Preventive Medicine/ Graduate School of Health Science Business Convergence, Chungbuk National University, Cheongju, Korea; 12 Department of Public Health and Preventive Medicine, Chungbuk National University Hospital, 776 1sunhwan-ro, Seowon-gu, Cheonju, 28644 Korea

**Keywords:** Prostatic neoplasms, Prostatectomy, Radiotherapy, Costs, National Health Insurance

## Abstract

**Background:**

A complete enumeration study was conducted to evaluate trends in national practice patterns and direct medical costs for prostate cancer (PCa) in Korea over a 10-year retrospective period using data from the Korean National Health Insurance Service.

**Methods:**

Reimbursement records for 874,924 patients diagnosed between 2002 and 2014 with primary PCa according to the International Classification of Disease (ICD) 10th revision code C61 were accessed. To assess direct medical costs for patients newly diagnosed after 2005, data from 68,596 patients managed between January 2005 and 31 December 2014 were evaluated.

**Results:**

From 2005 to 2014, the total number of PCa patients showed a 2.6-fold increase. Surgery and androgen deprivation therapy were the most common first-line treatment, alone or within the context of combined therapy. Surgery as a monotherapy was performed in 23.5% of patients in 2005, and in 39.4% of patients in 2014. From 2008, the rate of robot-assisted RP rose sharply, showing a similar rate to open RP in 2014. Average total treatment costs in the 12 months post-diagnosis were around 10 million Korean won. Average annual treatment costs thereafter were around 5 million Korean won. Out-of-pocket expenditure was highest in the first year post-diagnosis, and ranged from 12 to 17% thereafter.

**Conclusions:**

Between 2005 and 2014, a substantial change was observed in the national practice pattern for PCa in Korea. The present data provide a reliable overview of treatment patterns and medical costs for PCa in Korea.

## Background

Prostate cancer (PCa) is one of the most commonly diagnosed malignancies, and the sixth leading cause of cancer-related death, in men worldwide, particularly in developed countries [1, 2]. Between 2000 and 2010, the ranking/prevalence of PCa in the total Korean population increased from 14th/9881 to 7th/36,105 [3]. As a result of this increasing prevalence, the economic burden of PCa in Korea has shown a progressive increase, similar to that observed in other developed countries. In the US, the annual cost of PCa management totals several billion dollars [4]. Research in Korea has shown that direct treatment costs for PCa increased from $26 million in 2000 to $194 million in 2010 [5]. Reliable estimates of the medical cost of cancer management are essential to establish national priorities for resource allocation in health services [5–7]. At the macro-level, such estimates can provide references for budget planning, and are necessary for monitoring the flow of national health expenditure [8]. Despite the increasing burden of PCa, sparse nationwide data are available concerning the economic burden of PCa in the Korean population. Moreover, multiple treatment strategies with confirmed effectiveness for PCa are now available [9]. Increasingly, new technologies are being applied to treat PCa, with a rapid increase in the uptake of robotic-assisted laparoscopic radical prostatectomy (RARP) being observed [10]. Estimation of the influence of national practice patterns is required to elucidate the chronological change in the economic burden of PCa. To date, few studies have performed a comprehensive analysis of primary treatment patterns for PCa in Korea, with only a small number of sample survey studies having been reported. To document national practice patterns and medical costs in the entire Korean PCa population, a complete enumeration study is warranted.

The Korean government operates a mandatory national health insurance service (NHIS), which covers approximately 98% of the Korean population [11]. The NHIS also collects information concerning the residual population, which includes medical aid beneficiaries. The Korean National Health Insurance System (KNHI) is thus a nationwide population-based database, which contains comprehensive treatment-related information from the entire Korean population [12]. Using this unique database, the present study estimated chronological trends in national practice patterns among Korean PCa patients. Annual medical costs incurred through diagnosis, total medical costs, and out-of-pocket expenditure were also estimated.

## Methods

### Data sources and identification of prostate cancer patients

This study used National Health Information Database (NHIS-2017-4-029) made by Korean National Health Insurance Service (NHIS) [13, 14]. Because almost all of the payments were based on fee-for-service, National Health Insurance (NHI) claim data contains a specific disease code and all data necessary for reimbursement, including patient socio-demographic information such as sex, age, health insurance premiums, residential area, comorbid diseases, diagnostic tests, procedures, and prescriptions provided, and outcomes (deaths). Claim data from 874,924 patients with code C61, indicating PCa according to the International Classification of Diseases, 10th edition, Clinical Modification (ICD-10-CM) from 2002 to 2015 were screened. Patients had to fulfill the following requirements to be enrolled in the study: (1) undergone primary active treatment for PCa from 2003 onwards (*n* = 83,405); (2) availability of claim information for a minimum of 2 years before and 1 year after the primary treatment. Therefore, the final study group comprised 68,596 patients.

### Operational definition of primary treatment types and direct medical costs

Primary treatment methods included surgery, androgen deprivation therapy (ADT), and radiation therapy (RT). Surgical techniques included radical prostatectomy (RP) and RARP. The KNHI reimbursement codes for RP were ‘R3950’, ‘R3960’, and ‘RZ512’. RARP is not reimbursed through the KNHI, and thus cannot be identified via this coding system. RARP was therefore defined operationally as the absence of a surgery code despite the presence of a general anesthesia code (‘L1211’) and a postoperative pathology examination code (code ‘C5500’, ‘C5500’, ‘C5501’, ‘C5502’, ‘C5503’, ‘C5504’, ‘C5505’, ‘C5506’, ‘C5507’, ‘C5508’, ‘C5509’, ‘C5911’, ‘C5912’, ‘C5913’, ‘C5914’, ‘C5915’, ‘C5916’, ‘C5917’, ‘C5918’, or ‘C5919’). Primary ADT refers to both surgical orchiectomy and medical castration. Medical castration methods include luteinizing hormone-releasing hormone (LHRH) agonist only, anti-androgen only, and maximal androgen blockade (MAB).

In the present study, ‘cost’ refers to direct medical costs, excluding indirect costs secondary to the PCa and out-of-pocket expenditure not covered by the health insurance premium. All NHIS claim files, including inpatient, outpatient, and outpatient prescriptions, were used to estimate the total medical care costs for each PCa patient. For each medical procedure, an estimate was made of the average annual medical cost, the proportion of patient’s co-payments, and the duration of hospitalization or outpatient follow-up from 1 year before, and up to the 10th year after diagnosis. Based on information provided by Intuitive Surgical Korea Ltd. (Seoul, Korea), the cost of RARP was assumed to be 7 million won. The Hospital Input Price Index (unit price per point of the relative value scales) was used to adjust for inflation during the study period. All cost estimates are reported in 2015 Korean won.

### Other variables and statistical analysis

Patient sociodemographic parameters included age, income class, and residential area. Patients were divided into five categories according to age at diagnosis (< 50, 50–64, 65–74, and ≥ 75 years). Based on income levels indicated in the KNHI, patients were classified according to insurance premium categories below the poverty line (lowest) or quintile (I, II, III, IV, and V [highest]). KNHI contribution was used as a proxy measure for actual household income, since it is calculated on the basis of the income, property, and private car tax level of the respective household [15]. Residential area was divided into three categories (metropolitan, urban, and suburban/rural), according to the Korean ZIP code. To categorize comorbidity, the Charlson comorbidity index was used. This is a single index of comorbidity burden, which was developed to assess the relative risk of a comorbid condition and determine patient outcome following critical illness. The present cohort was grouped into four categories on the basis of this index score: 0, 1–2, 3–4, and ≥ 5 (most severe) [16]. Descriptive statistics were used to characterize treatment patterns and medical costs according to sociodemographic factors. To assess the strength of the ordinal relationship between total treatment costs (or the proportion of out-of-pocket expenditures) and time since the second year after diagnosis, we checked Kendall’s Tau-b correlation as a nonparametric measure of ordinal association in 2005. We also performed regression on total cost during the first year of primary treatment to estimate the association between total costs and demographic and clinical characteristics of patients. To correct for the skewed distribution of the medical cost data, we used the log-transformed costs as the outcome variable. All analyses were performed using SAS software (version 9.4).

## Results

### Demographic trends for prostate cancer in Korea from 2005 to 2014

The number of PCa patients in 2005 was 3548. This showed a steady annual increase across the 10 year study period, with an approximately 2.6-fold increase in the total number being observed in 2014. However, the rate of increase has steadily declined over the 10 year study period. The number of PCa patients below the age of 50 years decreased from 1.1 to 0.8%, while the number of those aged 75 years or older increased from 31.4 to 32.4%. However, no significant change in the mean age was observed over the 10 year study period. In 2005, metropolitan, urban, and rural areas of residence were reported for 59.9, 24.6, and 14.8% of the patients, respectively. This distribution remained unchanged over the 10 year study period. The number of patients living below the poverty line decreased from 17.7% in 2005 to 5.0% in 2014. Over the 10 year study period, the proportion of patients with no comorbidity (Charlson comorbidity score = 0) decreased, and the proportion of patients with comorbidity (Charlson comorbidity score ≥ 3) increased (Table 1).

**Table 1:** Demographics trend of prostate cancer in Korea from 2005 to 2014

Characteristic	2005	2006	2007	2008	2009	2010	2011	2012	2013	2014
Total (N)	3548	4313	5080	6225	6873	7262	8449	8825	8929	9092
Age (yr)										
Mean ± SD	70.3 ± 8.5	70.1 ± 8.5	70.0 ± 8.3	69.9 ± 8.3	69.9 ± 8.2	69.9 ± 8.3	70.0 ± 8.2	70.3 ± 8.3	70.1 ± 8.2	70.5 ± 8.3
< 50	39 (1.1)	56 (1.3)	56 (1.1)	65 (1.0)	73 (1.1)	83 (1.1)	82 (1.0)	67 (0.8)	73 (0.8)	75 (0.8)
50–64	809 (22.8)	992 (23.0)	1153 (22.7)	1446 (23.2)	1497 (21.8)	1607 (22.1)	1932 (22.9)	2017 (22.9)	2064 (23.1)	2006 (22.1)
65–74	1585 (44.7)	1958 (45.4)	2379 (46.8)	2966 (47.6)	3333 (48.5)	3482 (47.9)	3939 (46.6)	3957 (44.8)	4072 (45.6)	4062 (44.7)
≥ 75	1115 (31.4)	1307 (30.3)	1492 (29.4)	1748 (28.1)	1970 (28.7)	2090 (28.8)	2496 (29.5)	2784 (31.5)	2720 (30.5)	2949 (32.4)
Residential area										
Metropolitan	2124 (59.9)	2521 (58.5)	2916 (57.4)	3613 (58.0)	4040 (58.8)	4223 (58.2)	4971 (58.8)	5193 (58.8)	5311 (59.5)	5150 (56.6)
Urban	873 (24.6)	1074 (24.9)	1431 (28.2)	1695 (27.2)	1829 (26.6)	1954 (26.9)	2241 (26.5)	2278 (25.8)	2358 (26.4)	2515 (27.7)
Rural	524 (14.8)	583 (13.5)	733 (14.4)	917 (14.7)	1004 (14.6)	1085 (14.9)	1234 (14.6)	1354 (15.3)	1238 (13.9)	1368 (15.0)
Unknown	27 (0.8)	135 (3.1)	–	–	–	–	3 (0.0)	–	22 (0.2)	59 (0.6)
Income level, quintiles										
Below poverty line (lowest)	628 (17.7)	615 (14.3)	339 (6.7)	356 (5.7)	407 (5.9)	438 (6.0)	521 (6.2)	493 (5.6)	572 (6.4)	459 (5.0)
I	326 (9.2)	451 (10.5)	571 (11.2)	673 (10.8)	808 (11.8)	863 (11.9)	967 (11.4)	975 (11.0)	1026 (11.5)	1103 (12.1)
II	337 (9.5)	380 (8.8)	581 (11.4)	614 (9.9)	720 (10.5)	710 (9.8)	847 (10.0)	856 (9.7)	921 (10.3)	946 (10.4)
III	396 (11.2)	501 (11.6)	731 (14.4)	832 (13.4)	918 (13.4)	994 (13.7)	1128 (13.4)	1215 (13.8)	1184 (13.3)	1228 (13.5)
IV	631 (17.8)	763 (17.7)	925 (18.2)	1214 (19.5)	1240 (18.0)	1377 (19.0)	1621 (19.2)	1657 (18.8)	1724 (19.3)	1776 (19.5)
V (highest)	1230 (34.7)	1603 (37.2)	1933 (38.1)	2536 (40.7)	2780 (40.4)	2880 (39.7)	3365 (39.8)	3629 (41.1)	3502 (39.2)	3580 (39.4)
Charlson comorbidity index										
Mean ± SD	3.9 ± 3.6	3.9 ± 3.4	3.8 ± 3.4	4.0 ± 3.5	4.2 ± 3.5	4.2 ± 3.4	4.2 ± 3.5	4.4 ± 3.5	4.4 ± 3.5	4.4 ± 3.6
0	616 (17.4)	584 (13.5)	607 (11.9)	701 (11.3)	673 (9.8)	682 (9.4)	741 (8.8)	747 (8.5)	748 (8.4)	752 (8.3)
1–2	1030 (29.0)	1394 (32.3)	1738 (34.2)	1984 (31.9)	2117 (30.8)	2256 (31.1)	2648 (31.3)	2551 (28.9)	2609 (29.2)	2648 (29.1)
3–4	637 (18.0)	804 (18.6)	1073 (21.1)	1357 (21.8)	1586 (23.1)	1703 (23.5)	1966 (23.3)	1990 (22.5)	2067 (23.1)	2159 (23.7)
≥ 5	1265 (35.7)	1531 (35.5)	1662 (32.7)	2183 (35.1)	2497 (36.3)	2621 (36.1)	3094 (36.6)	3537 (40.1)	3505 (39.3)	3533 (38.9)

*SD* Standard deviation

### National practice patterns for prostate cancer in Korea from 2005 to 2014

**Fig. 1:**
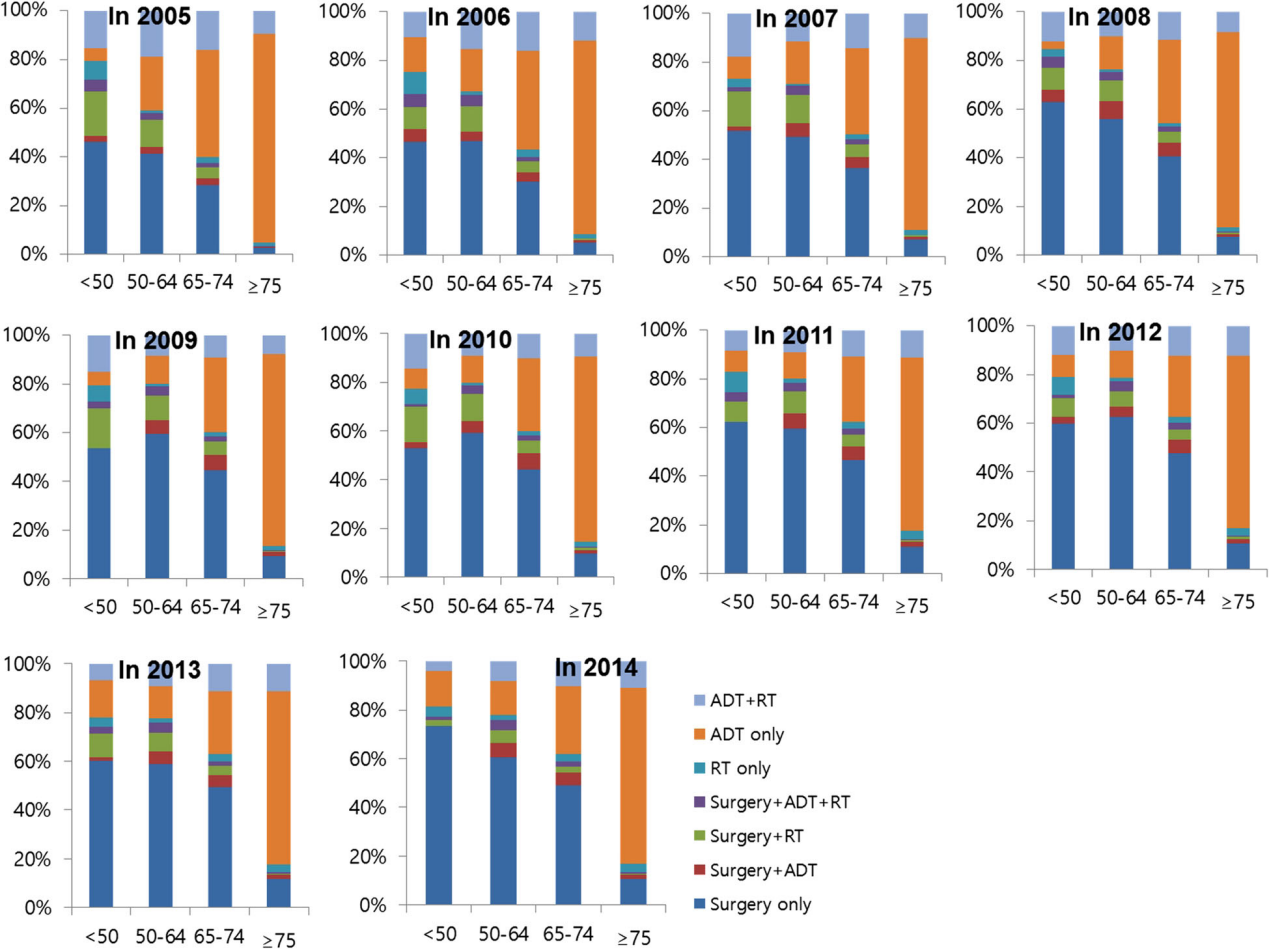
Trends in primary treatment for prostate cancer in Korea between 2005 and 2014 according to age group. ADT, androgen deprivation therapy; RT, radiotherapy

Between 2005 and 2014, a significant change was observed in national practice patterns for PCa in Korea. In 2014, surgery and ADT were the most common first-line treatment, with 47.9% of patients undergoing surgery (represented a 16% increase in comparison with 2005) and 54.9% of patients receiving ADT (represented a 14.9% decrease in comparison with 2005), alone or within the context of combined therapy. Surgery as a monotherapy was performed in 23.5% of patients in 2005, and in 39.4% of patients in 2014. While radiotherapy (RT) monotherapy showed an almost 2-fold increase during the 10 year study period (1.9% in 2005 vs. 3.2% in 2014), the use of RT as part of a collaborative, multimodal approach showed a slight decrease (20.9% in 2005 vs. 14.4% in 2014). ADT monotherapy decreased over time (51.6% in 2005 vs. 39% in 2014). The majority of these patients received MAB or LHRH agonist monotherapy. In 2014, surgical castration (orchiectomy) was rarely performed. A significant change was also observed across the 10 year study period in terms of surgical procedure. Here, the most noticeable change was the increased application of RARP. The use of RARP rose sharply after 2008, showing a similar rate to open RP in 2014 (Table 2). Analysis of treatment pattern according to age group revealed an increased proportion of surgery in all age groups, particularly in patients over 75 years of age (Fig. 1).

**Table 2:** Trends in national practice patterns of prostate cancer in Korea from 2005 to 2014

Characteristic	2005	2006	2007	2008	2009	2010	2011	2012	2013	2014
Total (N)	3548	4313	5080	6225	6873	7262	8449	8825	8929	9092
Radical surgery										
RP	1102 (97.4)	1478 (94.4)	1812 (85.0)	1774 (61.8)	1933 (56.6)	1893 (52.5)	2416 (56.6)	2432 (55.7)	2478 (55.7)	2217 (50.9)
RARP	29 (2.6)	88 (5.6)	320 (15.0)	1098 (38.2)	1480 (43.4)	1710 (47.5)	1850 (43.4)	1935 (44.3)	1973 (44.3)	2139 (49.1)
ADT										
Orchiectomy	174 (7.0)	117 (4.1)	90 (2.9)	79 (2.2)	67 (1.8)	41 (1.0)	38 (0.8)	32 (0.7)	21 (0.4)	28 (0.6)
LHRH agonist only	769 (31.1)	887 (31.2)	1093 (34.8)	1155 (31.5)	1243 (32.6)	1231 (30.8)	1602 (35.6)	1655 (34.5)	1596 (33.6)	1740 (34.8)
MAB	1322 (53.4)	1613 (56.7)	1791 (57.1)	2164 (58.9)	2228 (58.5)	2383 (59.6)	2531 (56.3)	2714 (56.6)	2760 (58.2)	2874 (57.5)
Anti-androgen only	210 (8.5)	230 (8.1)	163 (5.2)	274 (7.5)	271 (7.1)	345 (8.6)	328 (7.3)	396 (8.3)	368 (7.8)	352 (7.0)
Primary treatment										
Surgery only	835 (23.5)	1153 (26.7)	1576 (31.0)	2187 (35.1)	2592 (37.7)	2738 (37.7)	3314 (39.2)	3478 (39.4)	3595 (40.3)	3585 (39.4)
Surgery+ADT	74 (2.1)	124 (2.9)	186 (3.7)	297 (4.8)	336 (4.9)	334 (4.6)	387 (4.6)	360 (4.1)	350 (3.9)	368 (4.0)
Surgery+RT	170 (4.8)	208 (4.8)	274 (5.4)	278 (4.5)	358 (5.2)	400 (5.5)	398 (4.7)	327 (3.7)	333 (3.7)	225 (2.5)
Surgery+ADT + RT	52 (1.5)	81 (1.9)	96 (1.9)	110 (1.8)	127 (1.8)	131 (1.8)	167 (2.0)	202 (2.3)	173 (1.9)	178 (2.0)
RT only	68 (1.9)	105 (2.4)	93 (1.8)	88 (1.4)	114 (1.7)	124 (1.7)	238 (2.8)	223 (2.5)	256 (2.9)	288 (3.2)
ADT only	1831 (51.6)	2011 (46.6)	2216 (43.6)	2613 (42.0)	2745 (39.9)	2820 (38.8)	3044 (36.0)	3185 (36.1)	3271 (36.6)	3544 (39.0)
ADT + RT	518 (14.6)	631 (14.6)	639 (12.6)	652 (10.5)	601 (8.7)	715 (9.8)	901 (10.7)	1050 (11.9)	951 (10.7)	904 (9.9)

*RP* Radical prostatectomy, *RARP* Robotic-assisted laparoscopic radical prostatectomy, *ADT* Androgen deprivation therapy, *LHRH* Luteinizing hormone-releasing hormone, *MAB* Maximal androgen blockade, *RT* Radiotherapy

### Direct medical cost of prostate cancer in Korea from 2005 to 2014

Total treatment costs in the first 12 months post-diagnosis were around 10,719,000 Korean won (KRW). Average annual treatment costs thereafter were around 5,320,000 KRW. Out-of-pocket expenditure was highest in the first year after diagnosis (16.1% in 2005 and 22.1% in 2014), and ranged from 12 to 17% thereafter (Table 3). There were a significant negative monotonic association between total costs and time (coefficient − 0.78, *P* value 0.003 in 2005) and positive relation between the proportion of out-of-pocket expenditures and time (coefficient 0.63 *P* value 0.02 in 2005).

**Table 3:** Direct medical costs and proportion of patient’s co-payment for prostate cancer in Korea from 2005 to 2014

Characteristic	2005	2006	2007	2008	2009	2010	2011	2012	2013	2014
1 yr. before	2956 (27.8)	3307 (23.0)	3532 (22.0)	3793 (22.2)	3854 (22.3)	4020 (21.0)	4206 (20.4)	4183 (20.2)	4058 (20.1)	4141 (19.7)
1st yr	9077 (16.1)	9890 (13.7)	10,088 (16.7)	10,448 (24.0)	10,752 (24.6)	11,025 (23.6)	11,568 (22.9)	11,305 (22.0)	11,299 (22.0)	11,741 (22.1)
2nd yr	6220 (13.9)	6086 (14.6)	5897 (15.1)	5802 (13.7)	5831 (12.7)	5944 (12.1)	5563 (12.8)	5544 (12.9)	5793 (12.4)	
3rd yr	5711 (14.9)	5862 (15.2)	5534 (14.3)	5650 (13.0)	5521 (12.9)	5178 (13.4)	5191 (13.4)	5393 (13.3)		
4th yr	5473 (15.7)	5534 (14.3)	5261 (13.5)	5273 (13.4)	4985 (13.7)	5023 (13.9)	5152 (13.8)			
5th yr	5491 (14.5)	5529 (13.8)	5098 (13.7)	5034 (14.2)	4970 (14.1)	5140 (14.3)				
6th yr	5437 (14.8)	5211 (15.2)	4767 (15.6)	4603 (16.5)	4900 (15.7)					
7th yr	5324 (14.8)	5070 (15.2)	4800 (16.0)	4816 (16.2)						
8th yr	4952 (16.1)	4847 (15.8)	4912 (15.9)							
9th yr	4959 (16.4)	5057 (15.9)								
10th yr	5079 (16.4)									

Average annual payments from 1 yr. prior to primary treatment to 10th yr. thereafter (1000 KRW); *KRW* Korean won

**Fig. 2:**
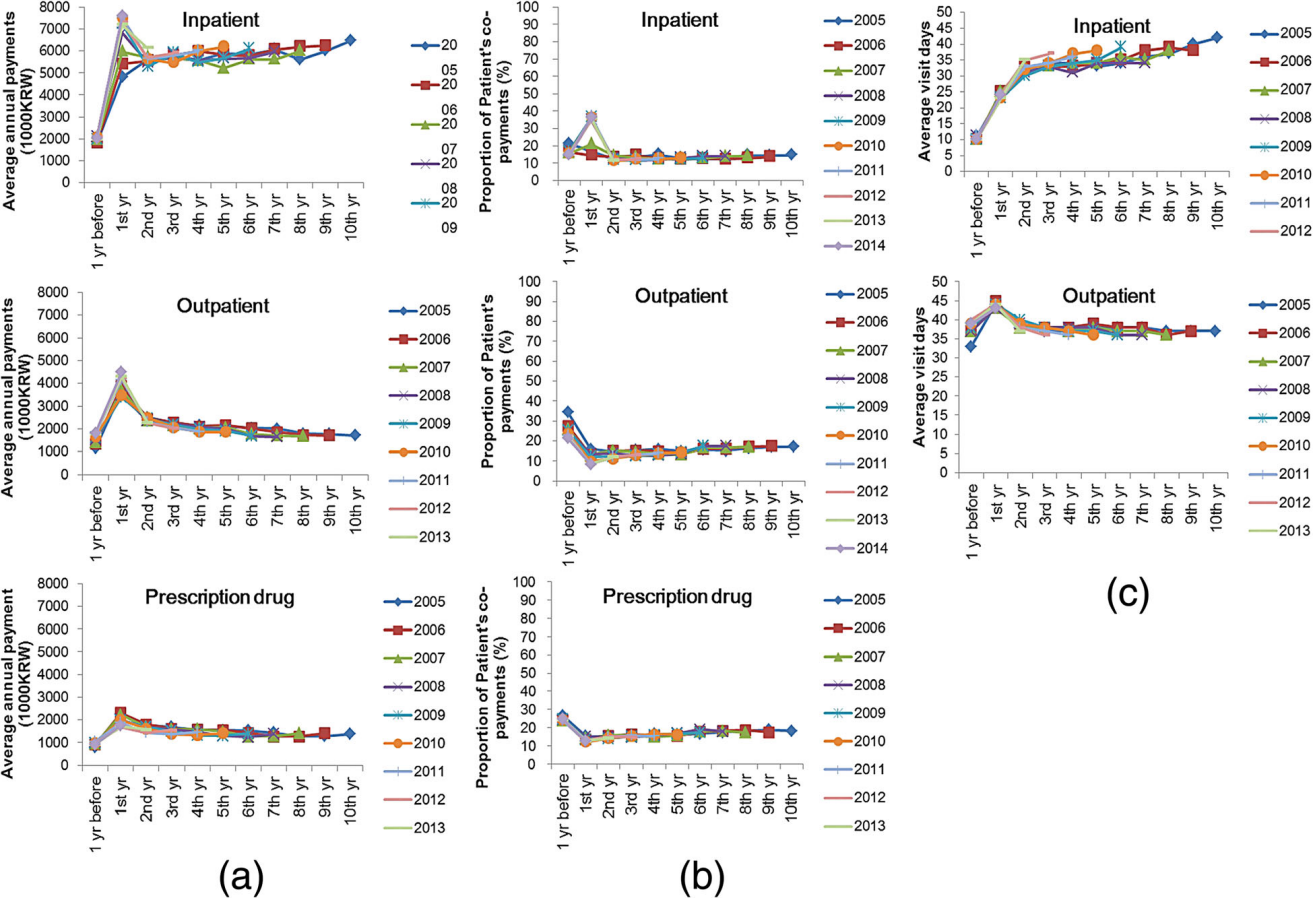
Average annual medical costs (**a**), proportion of patient’s co-payments (**b**), and duration of hospitalization or outpatient attendance (**c**) from the first year to 10 years after prostate cancer diagnosis

Prior to 2008, the total medical cost of hospitalization remained stable in the first year of diagnosis and thereafter, However, after 2008, costs in the first year of diagnosis increased. The cost of outpatient visits and drug prescriptions peaked in the 12 months post-diagnosis, and remained stable or showed a slight decline thereafter (Fig. 2a). Prior to 2008, out-of-pocket expenditure decreased from the first year and thereafter. After 2008, high out-of-pocket expenditure was observed in the first year post-diagnosis. Out-of-pocket expenditure for outpatient visits and drug prescriptions was lower after PCa diagnosis than before PCa diagnosis (Fig. 2b). Over the 10 year study period, the average length of hospitalization for PCa management showed a steady increase. By contrast, outpatient visits tended to decrease, with the exception of the first year post-diagnosis (Fig. 2c).

**Fig. 3:**
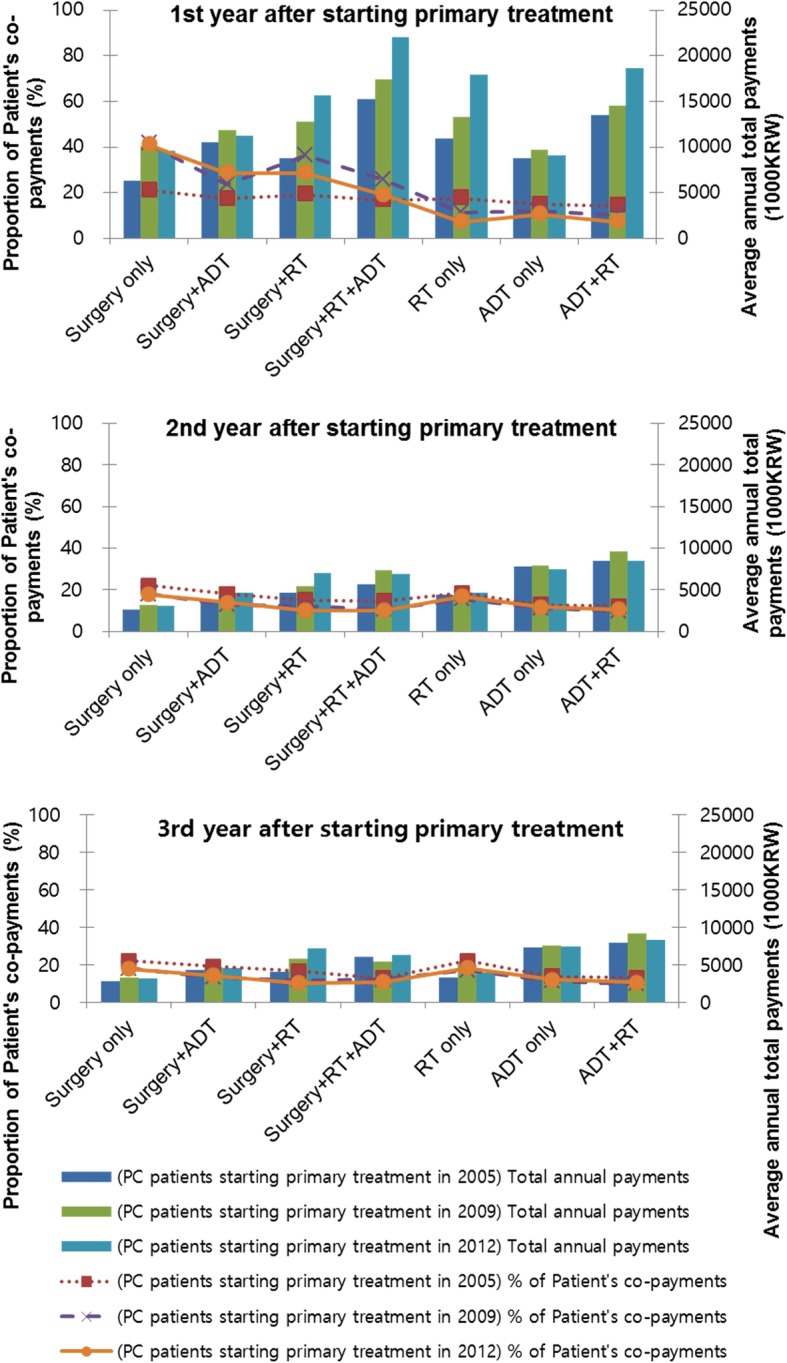
Average annual medical costs and proportion of patient’s co-payments according to primary treatment modalities from the first year to 3 years after prostate cancer diagnosis. PC, prostate cancer; ADT, androgen deprivation therapy; RT, radiotherapy

RT as monotherapy or as part of a collaborative, multimodal approach was the most expensive form of management. However, from 2008 onwards, out-of-pocket expenditure of patients in the 12 months post-diagnosis was highest for surgery (Fig. 3).

### Predictors of direct medical cost for prostate cancer during the first 12 months post-diagnosis

Older age was negatively associated with total treatment cost in the first 12 months post-diagnosis. Compared with patients below poverty line, those with insurance premium levels I and II were more likely to spend lower cost. Moreover, the presence of co-morbidity and multimodal approach were also associated with higher total cost (Table 4).

**Table 4:** Predictors of direct medical cost for prostate cancer during the first 12 months post-diagnosis

Variables	Unadjusted		Adjusted	
	Coefficient	*P* value	Coefficient	*P* value
Age, years	−0.0067	<.0001	−0.0015	<.0001
Income level, quintile				
Below poverty line (lowest)				
I	0.0012	0.9142	−0.0241	0.0144
II	0.0070	0.5280	−0.0306	0.0025
III	0.0297	0.0045	−0.0074	0.4403
IV	0.0267	0.0071	−0.0136	0.1357
V (highest)	0.0335	0.0003	0.0090	0.2886
Residential area				
Metropolitan				
Urban	0.0023	0.6586	0.0055	0.2516
Rural	−0.0092	0.16	0.0156	0.0092
Charlson comorbidity index				
0				
1–2	0.0639	<.0001	0.0715	<.0001
3–4	0.1377	<.0001	0.1572	<.0001
≥ 5	0.1791	<.0001	0.1957	<.0001
Primary treatment				
Surgery only				
Surgery+ADT	0.2549	<.0001	0.2570	<.0001
Surgery+RT	0.3352	<.0001	0.3403	<.0001
Surgery+ADT + RT	0.7928	<.0001	0.7961	<.0001
RT only	0.5093	<.0001	0.4997	<.0001
ADT only	−0.1133	<.0001	−0.1051	<.0001
ADT + RT	0.5354	<.0001	0.5421	<.0001
Adjusted-R			0.1812	

*ADT* Androgen deprivation therapy, *RT* Radiotherapy, Multivariable adjusted models were adjusted for age, income level, residential area, Charlson comorbidity index, and primary treatment type

## Discussion

The present complete enumeration study assessed trends in national practice patterns and direct medical costs for the entire PCa population in Korea between 2005 and 2014. The analyses revealed a marked change in national practice patterns. Out-of-pocket expenditure was highest in the first year post-diagnosis, and ranged from 12 to 17% thereafter with support from the KNHI program. However, from 2008 onwards, the sharp increase in the use of RARP resulted in higher total and out-of-pocket expenditure in the first 12 months post-diagnosis. Comparative-effectiveness research and new health technology assessments are therefore warranted for new procedures such as RARP.

The most important aspect of the present investigation study was the use of the complete enumeration study approach. Patients undergoing watchful waiting or active surveillance were not included. However, most PCa patients in Korea opt for active treatment, and thus the present data can be considered a reliable overview of national treatment patterns and medical costs for PCa in the Korean population. The major changes observed for PCa between 2005 and 2014 in Korea were (i) a pronounced increase in PCa prevalence; (ii) an expansion in the indication for surgical intervention, despite an increase in the proportion of elderly patients and comorbidities; and (iii) a sharp increase since 2008 in the use of RARP, which showed a similar rate to open RP in 2014. According to the respective KCCR annual report, in 2013 PCa accounted for 4.2% of all newly diagnosed cancers, with a crude incidence rate of 24.1 per 100,000 persons and an age-standardized rate of 15.0 per 100,000 persons. This rendered PCa the seventh most common cancer in the total population, and the fifth most common cancer among men [17]. Previous research shows that, between 1999 and 2013, age-standardized incidence rates for PCa showed a 10.7% annual percentage increase [18].

The preference toward the surgical management of PCa overlaps the increasing use of minimally invasive surgery using RARP. A large body of literature shows that RARP is an effective treatment for patients with localized PCa, and enthusiasm among surgeons for this technology has led to its widespread use [19, 20]. Research also shows that, in comparison with RP, the RARP procedure is associated with equivalent, or possibly superior, outcomes in terms of intraoperative and postoperative parameters such as continence, potency, and quality of life [21]. Thirty-six da Vinci robotic surgery platforms are currently in use throughout 30 hospitals in Korea [22]. According to a recent report, 24,207 patients (24,337 cases) underwent robotic surgery between 2005 and 2012, and the average annual growth rate for robotic surgery was 51.4% from 2005 to 2011 [22]. Recently, Park et al. reported the primary treatment patterns of 2702 Korean PCa patients between 2003 and 2013, using a 2% nationwide random sample of data from the KNHI [23]. The report demonstrated similar national practice patterns for Korean PCa as those found in the present study, and found that the use of RARP exceeded conventional RP in 2013 [23]. The present authors anticipate that RARP will remain a common procedure for the treatment of localized PCa. RARP will always be a more expensive procedure than conventional surgery due to the fixed capital and maintenance charges associated with the robotic system. At present, RARP was not found to be cost effective from a health care, economic standpoint compared with open approach although there was considerable uncertainty [24]. To prepare for the inevitable explosion in the demand for new technology, comparative-effectiveness research and careful consideration of the expansion of insurance coverage for RARP in Korea are warranted.

The present study involved a comprehensive examination of PCa burden in Korea during the period 2005 to 2014, as measured according to insurance premium/out-of-pocket expenditure and healthcare utilization. Previous study by Roehrborn et al. estimated of the socioeconomic burdens of PCa for different countries [4]. When costs were inflated to 2010 levels, the total estimated expenditure on PCa was mounted to 106.7–179.0 million euros in the European countries (UK, Germany, France, Italy, Spain and the Netherlands) and 9862 billion US dollars in 2006 [25, 26]. The mean annual costs per patient in the USA were $10,612 in the initial phase after diagnosis, $2134 for continuing care and $33, 691 in the last year of life [4, 26]. Data from government office statistics of Japan have shown that estimated cost of illness of PCa was 174.5 billion yen in 2002, 246.9 billion yen in 2005, 286.0 billion yen in 2008, and 307.3 billion yen in 2011 [27]. Recent studies examined cancer burden in Korea in the period 2000–2010 using national health insurance claims data. However, few recent studies have examined costs during phases of care beyond initial treatment and by stratifying for healthcare utilization [5]. Furthermore, estimation of out-of-pocket expenditure is more appropriate than estimation of the total treatment cost in terms of determining the economic burden of PCa patients. Korea has a single NHI program, which accounts for 97% of the population or approximately 50 million people [28]. The NHI operates on the basis of a coinsurance system, and statutory deductibles are dependent on the type of illness [11]. In September 2005, following the revision of the assessment exception criteria for partial co-payment, medical expenses for patients with cancer, cerebrovascular and cardiovascular disease, rare and incurable disease, or major burn injury were reduced [29]. For 5 years from the date of registration, cancer patients pay only 5% of the total medical costs for outpatient or inpatient care [29]. Although there was significant monotonic time trend in total costs and the proportion of patients’ copayment, the magnitude of Tau-b coefficients could be said to be small. While total treatment costs and out-of-pocket expenditure in the first year after diagnosis showed a slight increase, average treatment costs remained relatively stable thereafter although there was time trend. However, the non-insurance service such as selective medical fees or high-level hospital fees that are not covered by insurance premiums maintained a certain portion within total treatment cost of PCa. In particular, the rapid uptake from 2008 in the use of RARP resulted in an increase of more than 8% in out-of-pocket expenditure during the first 12 months post-diagnosis. The number of patients in the below poverty line category, whose medical expenses are paid in full, showed a gradual decrease over the study period. By contrast, patients in income level quartiles 1–3 still accounted for 40% of all Korean PCa cases in 2015. This suggests that the financial support system requires expansion, and strategies must be developed to end the association between illness and poverty in Korea.

The present study had two main limitations. First, the administrative KNHI database contains no information concerning clinical or pathological PCa stage. Thus, analyses of treatment patterns and medical cost according to stage were precluded. The recent increase in the detection of localized PCa through PSA-based screening may impact surgical indications and related costs. Second, the present cost estimates do not reflect indirect medical costs associated with factors such as transportation, caregiving, loss of patient/caregiver productivity/wages, and other cancer-related expenses. This may have led to an underestimation of the economic burden. A strength of the present investigation was that the study population represented all PCa patients who had undergone active treatments in Korea during the time period of interest. To our knowledge, this is the first report to document national practice patterns and medical costs for the entire Korean PCa population. The present data will facilitate the establishment of appropriate health strategies for PCa management in Korea.

## Conclusions

Between 2005 and 2014, a pronounced change was observed in the national practice pattern for PCa in Korea. While total treatment costs and out-of-pocket expenditure of PCa in the first year after diagnosis showed a slight increase, average treatment costs remained relatively stable thereafter although there was time trend. The present data provide a reliable overview of treatment patterns and medical costs for PCa in Korea.

## Data Availability

The data that supports the findings of this study is available from the Korean National Health Insurance Service (KNHIS), but restrictions apply to the availability of the data, which was used with permission for the current study and therefore not publicly available. Data is however available from the corresponding author upon reasonable request and with permission of KNHIS.

## Abbreviations

ADT: Androgen deprivation therapy
ICD: International Classification of Disease
KNHIS: Korean National Health Insurance Service
LHRH: Luteinizing hormone-releasing hormone
MAB: Maximal androgen blockade
PCa: Prostate cancer
RARP: Robotic-assisted laparoscopic radical prostatectomy
RP: Radical prostatectomy
RT: Radiotherapy
